# Multidimensional characterization, Landau levels and Density of States in epitaxial graphene grown on SiC substrates

**DOI:** 10.1186/1556-276X-6-141

**Published:** 2011-02-14

**Authors:** Nicolas Camara, Benoit Jouault, Bilal Jabakhanji, Alessandra Caboni, Antoine Tiberj, Christophe Consejo, Philipe Godignon, Jean Camassel

**Affiliations:** 1 Laboratoire Charles Coulomb, UMR 5221 CNRS-UM2, Place Eugène Bataillon, 34095 Montpellier Cedex 5, France; 2 CNM-IMB-CSIC - Campus UAB 08193 Bellaterra, Barcelona, Spain

## Abstract

Using high-temperature annealing conditions with a graphite cap covering the C-face of, both, on axis and 8° off-axis 4H-SiC samples, large and homogeneous single epitaxial graphene layers have been grown. Raman spectroscopy shows evidence of the almost free-standing character of these monolayer graphene sheets, which was confirmed by magneto-transport measurements. On the best samples, we find a moderate *p*-type doping, a high-carrier mobility and resolve the half-integer quantum Hall effect typical of high-quality graphene samples. A rough estimation of the density of states is given from temperature measurements.

## Introduction

It is now widely accepted that graphene-based devices are promising candidates to complement silicon in the future generations of high-frequency microelectronic devices. To this end, the most favourable technique to produce graphene for industrial scale applications seems to be epitaxial graphene (EG) growth. This can be done by chemical vapour deposition on a metal [1,2] or by heating a SiC wafer up to the graphitisation temperature [3-6]. In the first case, the disadvantage is the need to transfer the graphene film on an insulating wafer. In the second case, the SiC wafer plays the role of the insulating substrate without any need for further manipulation. Of course, to be suitable for the microelectronics industry, these EG layers must be continuous and homogeneous at the full wafer scale or, at least, on surfaces large enough to process devices.

On the Si-face of 6H or 4H SiC substrates, graphitisation at high temperature in an Ar atmosphere close to atmospheric pressure shows promising results for on-axis substrates. In this way, single-layer epitaxial graphene (SLEG) has already been grown at the full wafer scale [7,8] but an open issue remains the 6√3 SiC surface reconstruction which is a C-rich buffer monolayer on top of the SiC substrate. The first "real" graphene layer on top of this buffer layer is strained, not at all free-standing, strongly coupled to the C-rich buffer, heavily *n*-type doped, with a low-carrier mobility. On the contrary, on the C-face of the same SiC substrates, there is no need of a C-rich buffer layer at the interface before growing the first graphene layer [9-12]. In this way, the mobility could reach 30,000 cm^2^/V s in the work of Ref. [13].

For a long time, whatever the growth technique, the uniformity and quality of the EG was not good enough to find evidence of the so-called "half integer" quantum Hall effect (QHE). However, recently, large SLEG areas have been produced on the C-face of on-axis SiC substrates and, on such monolayer graphene, the carriers were holes with mobility close to the one found in mechanically exfoliated graphene films on SiO_2_/Si [14]. Consequently, the QHE could be demonstrated [15]. This shows clearly the advantage and quality of SLEG grown on the on-axis C-face of a SiC wafer over the on-axis Si-face. However, for further integration of graphene with current SiC technology, 8° off-axis substrates should be also considered since they constitute the standard in modern SiC industry [16].

In this work, we compare the results of graphene growth on semi-insulating, on axis and and 8° off-axis, 4H-SiC substrates. The quality, uniformity and size of the growth products will be compared using optical microscopy (OM), scanning electron microscopy (SEM), atomic force microscopy (AFM) and micro-Raman spectroscopy (μR). Then, Hall effect measurements will be done at different temperature in order to extract the density of states in the epitaxial monolayers.

### Graphene growth, microscopy and Raman studies

**Figure 1:**
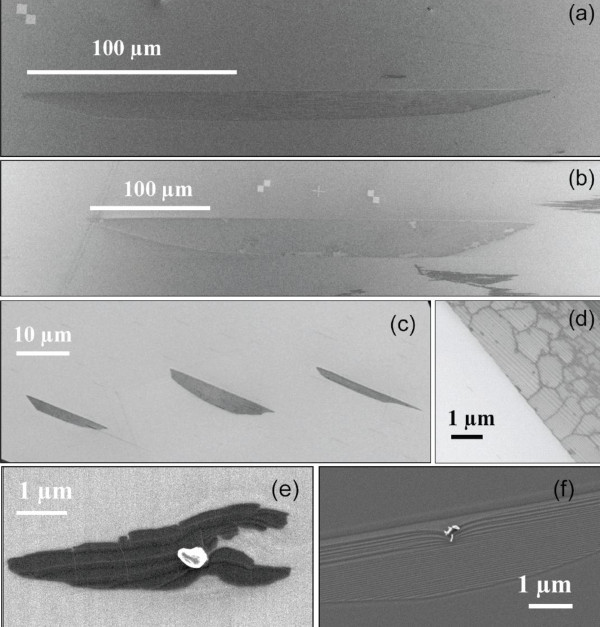
**SEM images of a monolayer graphene islands grown on the C-face of an 8° off-axis 4H-SiC substrate**. **(a, b) **Images of the largest homogeneous SLEG islands, **(c) **early growth, **(d) **zoomed image with visible wrinkles, **(e, f) **example of starting nucleation point by a surface defect with step bunching clearly visible in **(f)**.

To produce SLEG, in both cases of on axis and 8° off-axis SiC substrates, we used the recipes of Ref.[12]. On the on-axis material, this produces long, self-ordered, graphene ribbons which are typically 5 μm wide and several 100 μm long. This has been described at length in the work of Ref.[16]. On the off-axis substrates, this resulted also on SLEG islands but the morphology is completely different This is shown in Figure 1. Instead of narrow ribbons, after 30 min graphitisation at 1700°C, large SLEG islands can be obtained which can reach 300 μm long and 50 μm wide for the biggest ones. See Figure 1a and 1b. They can have a trapezoidal or triangular shape, see Figure 1a-c and 1f and, usually, nucleate from a defect on the surface. See Figure 1e and 1f. This may be either an unintentional particle remaining on the surface, a crystallographic defect such as a threading dislocation or a simple scratch made by a diamond tip. Whatever the origin, the growth starts from one nucleating centre and expands in a two-dimension carpet-like way. All resulting triangles are then self-oriented, with the longest side following the (11-20) plane direction.

**Figure 2:**
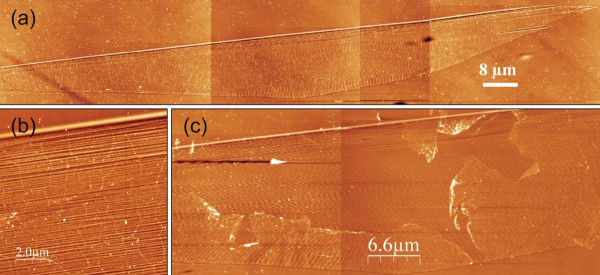
**AFM images of continuous and almost free standing monolayer graphene islands grown on the C-face of an 8° off-axis 4H-SiC substrate**. **(a) **at a large scale, the zoom in **(b) **showing the wrinkle and the step bunched character of the SiC surface below and **(c) **a layer scratched by an AFM tip.

In Figure 2a we show a typical AFM image of such a SLEG islands. When zooming, wrinkles become clearly visible in Figure 2 and show evidence of the continuity and strain-free character of the monolayers. Below the graphene islands, the step-bunched areas of the SiC surface are also clearly visible in both SEM and AFM pictures. The corresponding terraces are typically 100 nm wide and less than 2 nm high. A last evidence of the fact that the first layer of graphene is not coupled with the substrate and continuous despite the step-bunched surface is the facility with which we can remove the SLEG layer with an AFM tip. The result presented with the AFM picture of Figure 2c demonstrates the almost free-standing and continuous character of the grown SLEG.

Tens of similar monolayer islands grown on, both, on axis and off-axis substrates were probed by Raman spectroscopy. We used the 514 nm laser line of an Ar-ion laser for excitation and got very similar features. At the micrometer size, all spectra reveal that the islands are of the same nature and very homogeneous. First, the D-band, which usually indicates the presence of disorder or edges defects, is very weak and the Raman signature is extremely close to the one found for exfoliated graphene on SiO_2_/Si [11]. Second, the 2D-band appears at low frequency (2685 cm^-1^) which is strong evidence that there is no strain at the layer to substrate interface (i.e. almost a free-standing SLEG layer). Third, this 2D-band can be fitted with a single Lorentzian shape with a FWHM of 30 cm^-1 ^[17]. Fourth, the ratio *I*_2D_/*I*_G _between the integrated intensities of the 2D-band and the G-band is high, which suggests weak residual doping in the order of 3 to 6 × 10^12 ^cm^-2 ^[18]. Altogether, these Raman and microscopy measurements tend to demonstrate the almost free-standing low-doped and continuous character of the grown layers [12,19].

### Electrical transport measurements

**Figure 3:**
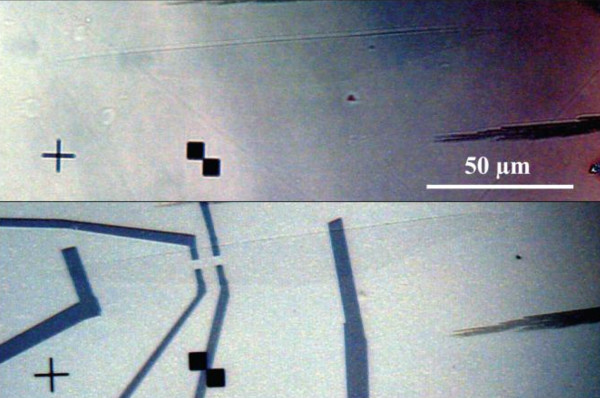
**Optical microscopy of a SLEG grown on 8° off-axis semi-insulating SiC substrate**. **(a) **before contact and **(b) **after contacting in a Hall Bar configuration for Hall Effect measurement.

Gold alignment marks were used to select some SLEG position by OM. Then, they were contacted by e-beam lithography and subsequent deposition of a contact layer made of Cr/Au in Hall bar configuration. A typical example is shown in Figure 3.

Then transport measurements were done at low temperature on the different samples, using a maximum magnetic field of 13.5 T. The contact geometry allowed simultaneous measurement of, both, the longitudinal and transverse voltages with the current flowing between two injection contacts at the flake extremities. In both series of samples, from the sign of the Hall voltage, we found that the carriers were holes (in agreement with other results published on the C-face [13,14]). The holes concentration ranged from 1 × 10^12 ^to 1 × 10^13 ^cm^-2 ^at low temperature, with a weak temperature dependence.

**Figure 4:**
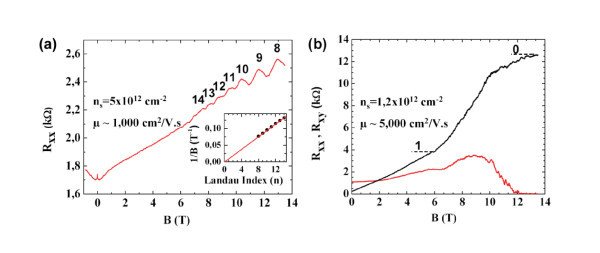
**Typical magnetoresistance measurements for low doped and highly doped epitaxial graphene-based Hall Bars**. **(a) **Longitudinal resistance of highly *p*-type doped epitaxial monolayer versus the magnetic field *B*, measured at 1.6 K. The resistance increases linearly with *B *with the superimposed SdH oscillations clearly resolved. Index of Landau levels (8-14) is also reported. Inset: the Landau plot indicates a phase equal to 0°, as expected for Dirac electrons. **(b) **Longitudinal and transverse resistance of low *p*-type doped epitaxial monolayer versus applied magnetic field *B*, at *T *= 1.6 K. The Hall resistance approaches the integer plateau *R*_*xy *_~12.9 kΩ at *B *~13 T. The second plateau at 4 kΩ is hardly visible.

For carrier concentrations larger than 3 × 10^12 ^cm^-2^, **no **QHE could be detected and only Shubnikov-de Haas (SdH) oscillations were found. This is shown in Figure 4 for an off-axis sample and, as usual, the plot of the inverse field at which the oscillations maxima occur versus the Landau level index shows a clear linear dependence going down to the origin. This is the usual signature of the heavily doped graphene.

For the low doped layers, the transverse resistance exhibits now quantized Hall plateaus, clearly governed by the sequence *R*_*K*_/4(*N *+ 1/2) in which *R*_*K *_= *h*/*e*^2 ^is the Von Klitzing constant [20] and *N *= 0, 1, 2... As already known, this peculiar sequence of resistance values is the well-known quantum transport signature of the monolayer graphene Landau levels [14]. In Figure 4(b).we show the longitudinal and Hall resistance values for such a low-doped SLEG device with hole concentration *n*_s _= 1.2 × 10^12 ^cm^-2 ^and mobility μ ~5000 cm^2^/V s at *T *= 1.6 K. At *B *= 12 T, the longitudinal resistance cancels while the transverse resistance tends to 12.9 kΩ which is the expected value for the *N *= 0 plateau (*R*_*K*_/2).

**Figure 5:**
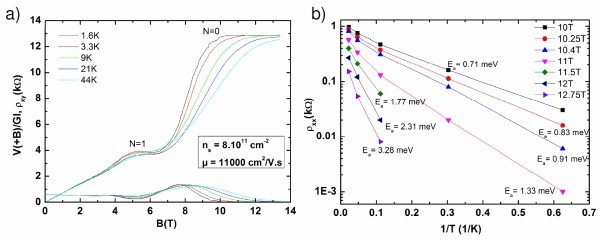
**Magnetoresistance measurements of the best sample at different temperatures**. **(a) **Longitudinal and transverse resistances of low *p*-type doped (*n*_s _= 8 × 10^11 ^cm^-2^) epitaxial monolayer versus applied magnetic field *B*, at different temperatures. **(b) **Temperature dependence of the resistivity *ρ*_*xx *_of a graphene ribbon at different magnetic field values close to the filling factor *v *= 3. The slope in the semilog scale gives the activation energy *E*_a_, which is the energy difference between the Fermi energy and the mobility edge of the second (*N *= 1) Landau level.

In Figure 5a, we present similar resistance measurements obtained with a lower doped sample with a hole concentration *n*_s _= 8 × 10^11 ^cm^-2 ^and a mobility *μ *~11,000 cm^2^/V s. The mobility is high enough and the concentration low enough to make the *N *= 0 and *N *= 1 plateaus well resolved and stable up to 13.5 T. The experimental results of Figure 5a have been obtained in a three probes configuration with low resistance contacts (40 Ω). The Hall resistance corresponds to the symmetric part of the signal: ρ_*xy *_~(*V*(*B*)+*V*(-*B*))/2*I*, where the voltage *V *is measured between a lateral probe and the current drain. At high magnetic fields, we identify *V*(+*B*)/*IG *as *ρ*_*xx*_, where *G~4 *is the geometric factor and *I *is the current.

The temperature dependence of *ρ*_*xx*_(*B*) is shown in Figure 5a, between 1.6 and 44 K. In this temperature range, an activated behaviour is found for the resistivity: *ρ*_*xx *_~exp(-*E*_a_/*k*_B_*T*) of the *N *= 0 plateau. This activation energy *E*_a _is the energy separation between the Fermi energy *E*_F _and the delocalised states of the *N *= 1 Landau level. In Figure 5b we plot the resistivities values ρ_*xx *_taken at different magnetic fields in the vicinity of the *R*_*K*_/2 plateau. The activation energy *E*_a _varies from 0.7 to 3.3 meV between *B *= 10 and 13 T, which remains much smaller than the distance between the first and the second Landau level (~120 meV at *B *= 10 T). This indicates that the Fermi energy is firmly pinned by localised states. *E*_a _has been calculated by taking into account only temperatures above 6 K. At lower temperatures, there is an additional contribution to the conductivity, which is visible in Figure 5b as a change in the slope. We attribute this additional contribution to hopping.

In principle, from the activation energy, we can reconstruct the density of state ρ(*E*). The filling factor is calculated from *B *= 10 to 13 T, each filling factor change Δν at a given magnetic field corresponding to a density variation Δ*n*_s _= *n*_s_Δν/ν. The Fermi energy shifts by Δ*E*_a _to compensate for the density variation and the mean value for the density of states at energy ~*E*_a _is given by ρ(*E*) = Δ*n*_s_/Δ*E*_a_.

**Figure 6:**
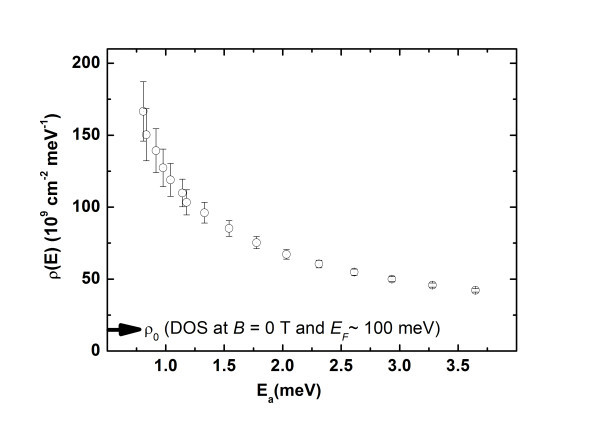
**Density of states *ρ*(*E*) as a function of the energy *E*_**a**_**. For comparison, the density of states without magnetic field at *E*_F _= 100 meV is indicated by an arrow.

Following this procedure, already used in the early times after the discovery of the integer QHE [21], we find the density of states plotted in Figure 6. The formation of the Landau level is evidenced as, when *E*_a _decreases, the density of states ρ(E) increases and becomes one order of magnitude larger than the density of states ρ_0_(E) without magnetic field at a comparable energy *E*_F _~100 meV: ρ_0_(E) ~15 × 10^9 ^cm^-2 ^meV^-1^. The shape of ρ(E) gives a rough upper bound of the half-width at half-maximum (HWHM) of the *N *= 1 Landau Level. We find HWHM ≤ 3 meV. This value is in good agreement with results obtained recently on EG by STM [22]. However, the extracted density is systematically larger than ρ_0 _over the whole investigated energy range. This observation, combined with the fact that hopping was neglected, indicates that more detailed investigations are still needed.

**Figure 7:**
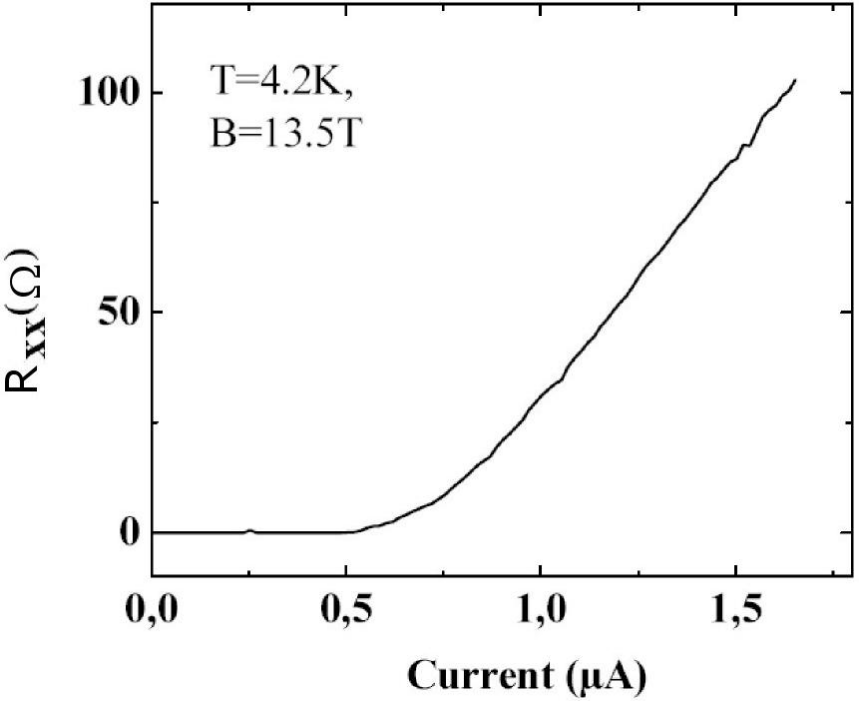
**Longitudinal resistance (in ohms) as a function of the injected current**. Breakdown of the quantization occurs at *I *= 0.5 μA.

Finally, since EG has recently been proposed for metrological application, we plot, in Figure 7, the longitudinal resistance as a function of the current at *B *= 13.5 T. This magnetic field is far from the filling factor υ = 2 and; therefore, the breakdown occurs at relatively low current: *I *= 0.5 μA, which corresponds to a current density *j *= 0.025 A/m. By comparison, for III-V heterostructures, critical current values of 1 A/m are reported.

## Conclusion

To summarize, we have shown the possibility to grow large islands of monolayer graphene on the C-face of on-axis and 8° off-axis commercial 4H-SiC wafers. The graphene layers are continuous, almost free-standing and show quantum transport properties comparable with high-quality, low-doped, exfoliated graphene. We show evidence of half-integer QHE specific of graphene monolayer and give a first estimate of the density of states in the magnetic field.

## Abbreviations

AFM: atomic force microscopy; EG: epitaxial graphene; HWHM: half-width at half-maximum; QHE: quantum Hall effect; SEM: scanning electron microscopy; SdH: Shubnikov-de Haas; SLEG: single-layer epitaxial graphene.

## Competing interests

The authors declare that they have no competing interests.

## Authors' contributions

NC and AC carried out the Graphene growth, the Hall Bars fabrication, the AFM, SEM and Raman characterisation. AT carried out the Raman investigation and interpretation. BJ, BJ and CC carried out the magnetotransport measurements. Finally PG and JC participated in the design and the coordination of this work. All authors read and approved the final manuscript.
